# 877. Diagnostic Values of Viral Target Enriched and Metagenomic NGS among Patients with Acute Undifferentiated Fever in Thailand

**DOI:** 10.1093/ofid/ofad500.922

**Published:** 2023-11-27

**Authors:** Julie Yamaguchi, Michael G Berg, Sonja Weiss, Pakpoom Phoompoung, Gavin Cloherty, Yupin Suputtamongkol

**Affiliations:** Abbott Labs, SCHAUMBURG, Illinois; Abbott Labs, SCHAUMBURG, Illinois; Abbott Labs, SCHAUMBURG, Illinois; Siriraj Hospital / Mahidol University, Thailand, Bangkok, Krung Thep, Thailand; Abbott, Abbott Park, Illinois; Mahidol University, Bangkok, Buriram, Thailand

## Abstract

**Background:**

Despite extensive lab testing, infectious agents were not detected in ∼50% of patients hospitalized for acute undifferentiated febrile illness (AUFI) at Siriraj Hospital, Bangkok, Thailand. Unbiased metagenomic Next Generation Sequencing (mNGS) enables detection of many microbes present in patient samples. Target enrichment for viruses in these libraries represents a highly sensitive and cost-effective approach for overcoming host background present in clinical specimens. Plasma collected from 440 patients with AUFI between 2014-2021 were analyzed by both viral target enrichment and mNGS to determine their diagnostic values for the identification of human pathogens.

**Methods:**

Benzonase-treated plasma was extracted on an Abbott *m*2000 instrument. mNGS libraries were prepared from ds-cDNA and hybridized to CVRP probes (Twist Biosciences) covering > 15,000 strains of vertebrate viruses. Target enriched libraries were run on a MiSeq; mNGS libraries were sequenced on a NextSeq. Reads were processed by the SURPI or DiVir pipelines and viral reads were mapped in the CLC Bio Genomics Workbench.

**Results:**

We identified viruses in 23% of our AUFI population using CVRP. mNGS increased viral detection to 26.8% and overall pathogen detection to 41.8%. Both methods identified relatively prevalent viruses such as Dengue, HIV-1, HBV, HAV and CHIKV, less common viruses such as Measles, HHV-6B, CMV, and PARV and emerging viruses such as SFTS virus with a high degree of correlation between NGS and clinical data. Bacterial pathogens identified by mNGS included *Rickettsia typhi*, *R. felis* and *Capnocytophaga canimorsus*. Identification of parasites *P. falciparum* and *P. vivax* was consistent with clinical presentations. mNGS failed to detect blood-culture positive patients with *Klebsiella pneumoniae*, *Pseudomonas aeruginosa,* and *Streptococcus gallolyticus* septicemia.

**Conclusion:**

CVRP and mNGS demonstrated that viral and rickettsial infections were important etiologies for AUFI in Thailand. This data supports the current recommendation of empirical therapy with ceftriaxone and doxycycline or azithromycin in severe or hospitalized AUFI. Implications for proper management of AUFI include lower rates of unnecessary testing and antimicrobial use.

**Disclosures:**

**Julie Yamaguchi, BS**, Abbott Labs: Patents|Abbott Labs: Employee|Abbott Labs: Stocks/Bonds **Michael G. Berg, PhD**, Abbott Laboratories: employee|Abbott Laboratories: Stocks/Bonds **Sonja Weiss, BS**, Abbott Laboratories: Employee|Abbott Laboratories: Stocks/Bonds **Pakpoom Phoompoung, MD**, Abbott Labs: Grant/Research Support|Abbott Labs: Mahidol University is a partner in the Abbott Pandemic Defense Coalition **Gavin Cloherty, PhD**, Abbott Labs: Patents|Abbott Labs: Employee|Abbott Labs: Stocks/Bonds **Yupin Suputtamongkol, MD**, Abbott Labs: Grant/Research Support|Abbott Labs: Mahidol University is partner in the Abbott Pandemic Defense Coalition

